# Subthalamic stimulation changes motor laterality in Parkinson’s disease

**DOI:** 10.1016/j.prdoa.2020.100081

**Published:** 2020-11-27

**Authors:** Zhengyu Lin, Chencheng Zhang, Bomin Sun, Dianyou Li

**Affiliations:** a Department of Neurosurgery, Ruijin Hospital Affiliated to Shanghai JiaoTong University School of Medicine, Shanghai, China; b Center for Functional Neurosurgery, Ruijin Hospital Affiliated to Shanghai JiaoTong University School of Medicine, Shanghai, China

**Keywords:** Parkinson’s disease, Subthalamic nucleus, Deep brain stimulation, Motor laterality

## Abstract

In this retrospective study, by assuming that the therapeutic impedances were equal to 1 kΩ, we found that the mechanism by which deep brain stimulation of the subthalamic nucleus (STN) changed the motor laterality in Parkinson's disease (PD) may not be related to the difference in weighted average of the total electrical energy delivered to each side of STN.

One of the prominent features of Parkinson’s disease (PD) is an asymmetrical distribution of motor symptoms at the onset and during the disease course [1]. Subthalamic deep brain stimulation (STN-DBS) altering the symptom laterality in PD had been suggested but underlying mechanisms remained further elucidated [2]. Here we observed that the bilateral STN-DBS changes the motor laterality of PD at the short-term follow-up, and this post-stimulating motor laterality alteration is probably not related to the stimulating intensity directly applied on each side of STN.

Sixteen PD patients undergoing STN-DBS in Ruijin Hospital (Shanghai, China) with full records of postoperative programming parameters [i.e., voltage (v), pulse width (pw), frequency (f)] were retrospectively studied. The median follow-up duration was 6 (range 6–10) months. The motor examination was performed and videotaped by one evaluator and independently scored (except for rigidity item) by one experienced movement disorder specialist blinded to medication and stimulator statuses. The sum score of the MDS UPDRS items 3.3–3.8 and 3.15–3.17 for each side of the body was calculated. The asymmetric index was defined as proposed by Ham et al. [3]. Six patients [38% (6/16)] demonstrated a change of motor symptom laterality after STN-DBS (Table 1). We wonder whether this post-stimulating motor laterality alteration could be associated with the asymmetry of total electrical energy delivered (TEED) [4] applied to each side of STN. By assuming that the therapeutic impedances were equal to 1 kΩ, we calculated the weighted average of the TEED for each side of STN, where the stimulating duration of each set of parameters served as the weighting factor. The stimulating asymmetry was defined as the (L − R)/(L + R) ratio ≥0.1 (left-STN-dominant stimulation) or ≤−0.1 (right-STN-dominant stimulation). No significant association was observed between preoperative motor laterality and average stimulating ‘intensity’ laterality (*p* = 0.433). Furthermore, there was no significant correlation between the direction of motor laterality change and average stimulating 'intensity' laterality (*p* = 0.131).

**Table 1:** Demographics and motor symptom laterality before and after subthalamic stimulation.

Number	Sex	Handedness	Age (y)	Disease duration (y)	Follow-up (m)	MDS UPDRS-III laterality						
						Baseline (Med off)			After STN-DBS (Med off/DBS off)			
						Left sum	Right sum	Motor laterality	Left sum	Right sum	Motor laterality	Stimulation asymmetry index
1	M	Right	67	10	10	14	12	Sym	17	16	Sym	0.10
2*	M	Right	66	12	6	21	25	Right	24	26	Sym	−0.16
3	M	Right	64	14	6	17	14	Sym	20	17	Sym	0.30
4*	M	Right	66	10	6	20	16	Left	24	21	Sym	−0.10
5	M	Right	69	7	6	19	21	Sym	21	19	Sym	−0.07
6	M	Right	67	9	6	16	21	Right	12	19	Right	0.25
7*	F	Right	53	14	6	10	10	Sym	19	15	Left	−0.06
8	M	Right	58	10	6	18	18	Sym	18	20	Sym	0.23
9	M	Right	59	6	8	24	22	Sym	21	20	Sym	−0.15
10*	M	Right	35	19	8	33	29	Left	22	23	Sym	0.12
11	F	Right	33	6	9	20	18	Sym	23	24	Sym	0.11
12	M	Right	36	11	6	25	25	Sym	29	29	Sym	0.12
13*	M	Right	46	9	6	12	12	Sym	22	18	Left	−0.08
14*	M	Right	33	6	6	25	23	Sym	23	11	Left	0.07
15	M	Right	59	12	6	14	9	Left	10	6	Left	−0.26
16	F	Right	64	12	6	18	12	Left	18	13	Left	−0.05
Mean (SD)			54.7 (13.5)	10.4 (3.5)	6.7 (1.3)							

*: PD Patients with motor laterality alteration after STN-DBS.

Abbreviation: MDS UPDRS-III = the Movement Disorder Society Unified Parkinson’s Disease Rating Scale Motor Part; Med off = Medication off condition; Med off/DBS off = Medication off/DBS off condition; M = Male; F = Female; Sym = symmetric.

A symmetric distribution of motor features has been reported to be associated with an unfavorable outcome of PD [5]. Although such correlation could be confounded by age and disease duration, the motor laterality change remains critical during disease progression of PD. In our study, three patients developed a symmetric distribution of motor patterns after STN-DBS. As the short-term follow-up reduces the possibility that the motor laterality alteration is related to disease progression, further studies are expected to investigate whether the early occurrence of postoperative motor laterality change may have a potentially greater impact on the postural and gait instability deterioration as well as the mental decline after long-term stimulation.

Our preliminary results also suggested that the discrepancy of stimulating intensity on each side of STN may probably not contribute to the postoperative motor laterality change. In fact, the mechanism of motor asymmetry in PD remains poorly understood [1]. Whether STN-DBS induces a symmetrically progressive loss of dopaminergic neurons or an asymmetrical neuroprotective effect needs further investigation.

One major limitation of this small-scale retrospective study is that we assumed the value of therapeutic impedance was the same across all subjects and parameters to calculate the TEED to each side of STN. Besides, the pre- and postoperative change of each sum score was relatively subtle, requiring a larger sample to draw the more conclusive findings. Further large-scale prospective studies are expected to investigate mechanisms by which DBS alters the distribution of motor features and the underlying clinical significance.

## Author contributions

1) Research project: A. Conception, B. Organization, C. Execution;
2) Statistical Analysis: A. Design, B. Execution;
3) Manuscript: A. Writing of the first draft, B. Review and Critique.

Zhengyu Lin: 1C, 2A, 2B, 3A

Chencheng Zhang: 1A, 1B, 2A, 3B

Bomin Sun: 3B

Dianyou Li: 1A, 1B, 3B

## Ethical compliance statement

The Ethical Committee of Ruijin Hospital approved the study protocol. All patients provided written informed consent for surgery and participation in the follow-up. We confirm that we have read the Journal’s position on issues involved in ethical publication and affirm that this work is consistent with those guidelines.

## Funding sources and conflict of interest

The authors declare that they have no conflicts of interest relevant to the manuscript.

## Financial disclosures

There are no financial conflicts of interest to disclose.
